# Analysis of Korean Peninsula Earthquake Network Based on Event Shuffling and Network Shuffling

**DOI:** 10.3390/e23091236

**Published:** 2021-09-21

**Authors:** Seungsik Min, Gyuchang Lim

**Affiliations:** 1 Department of Natural Science, Korea Naval Academy, Changwon 51704, Korea; 2 Kyungpook Institute of Oceanography, University of Kyungpook, Daegu 41566, Korea; econolim@daum.net

**Keywords:** Korean peninsula earthquake network, Abe and Suzuki method, event shuffling, network shuffling, complex network

## Abstract

In this work, a Korean peninsula earthquake network, constructed via event-sequential linking known as the Abe–Suzuki method, was investigated in terms of network properties. A significance test for these network properties was performed via comparisons with those of two random networks, constructed from two approaches, that is, *EVENT* (*SEQUENCE*) *SHUFFLING* and *NETWORK* (*MATRIX*) *SHUFFLING*. The Abe–Suzuki earthquake network has a clear difference from the two random networks. However, the two shuffled networks exhibited completely different functions, and even some network properties for one shuffled datum are significantly high and those of the other shuffled data are low compared to actual data. For most cases, the event-shuffled network showed a functional similarity to the real network, but with different exponents/parameters. This result strongly claims that the Korean peninsula earthquake network has a spatiotemporal causal relation. Additionally, the Korean peninsula network properties are mostly similar to those found in previous studies on the US and Japan. Further, the Korean earthquake network showed strong linearity in a specific range of spatial resolution, that is, 0.20°~0.80°, implying that macroscopic properties of the Korean earthquake network are highly regular in this range of resolution.

## 1. Introduction

The most powerful earthquake of magnitude 5.8 occurred in South Korea on 12 September 2016, and more than 200 aftershocks have since occurred. The earthquake with a magnitude of 5.0 or more on the Korean peninsula was rare because the Korean peninsula had been recognized as a safe zone for earthquakes. Although earthquakes are one of the most damaging natural disasters, observations and predictions of earthquakes are still very difficult. Thus, the studies on earthquakes have mainly focused on dealing with the estimating of geological mechanisms, discoveries of new crustal faults, or analyses of characteristics for seismic waves by seismologists. On the other hand, some studies on earthquakes have been carried out by architects and civil engineers because most earthquake damages are related to the strength of architecture.

In recent years, there have been many attempts to analyze and forecast the dynamical and topological properties of seismic events through statistical approaches including a new method of time series analysis termed natural time analysis [1] developed in the early 2000s, which enables the introduction of an order parameter of seismicity [2], the variability of which [3,4] reveals that there exist temporal correlations between the earthquake magnitudes [5,6]. Moreover, there have been new types of research combining the time series analysis and network analysis for spatiotemporal seismic data since the 2000s [7,8] as for example, upon combining natural time analysis with the network analysis [9], one can find an estimation of both the time of occurrence and the epicenter of an impending mainshock [10,11,12].

Network theory has been a great language to describe complex systems for several decades. Using the matrix and graph theory of mathematical languages, statistical physicists have tried to explain the characteristics of complex networks. Erdös and Rényi [13] proposed a random graph theory, which has developed a complex network theory. Watts and Strogatz [14] generated a random network and found the small-world property. Barabási and Albert [15] generated a random complex network and found the scale-free property. Moreover, Cohen and Havlin [16] revealed that the scale-free network has ultra-small properties in many cases. Nowadays, scientists use the complex network theory in physics, biology, geology, IT, economics, social sciences, and so on [17,18,19,20,21,22,23,24,25,26]. Some researchers may obtain global properties through complex network analyses, others may be interested in finding local properties, network stability, dynamics and evolution [27,28,29,30,31].

When a network analysis is performed for a number of time-series data, each time series is generally used as a node and a link between nodes is defined in various ways. In this case, the link is often made up of the correlation coefficient between two nodes. However, seismic data are spatiotemporal because it is not a time series that occurs continuously in a fixed space. Therefore, it is not easy to grasp the relation between two specific points. Abe and Suzuki constructed an earthquake network and analyzed the characteristics of the network by partitioning the survey area into grids, setting them as the nodes and establishing links between two grids of consecutive seismic observations [32]. Thereafter, a number of subsequent studies were performed analyzing the earthquake network constructed by their method.

In this paper, we present the geographical distributions and frequency trends observed in the Korean peninsula from 2005 to 2020 and introduce the Abe–Suzuki method in Section 2. Then, we establish the earthquake network and calculate the main network parameters such as characteristic path length, clustering coefficient, assortativity, and modularity in Section 3. Finally, the meaning of this study is summarized in Section 4 and discussed in Section 5.

## 2. Data and Methods

### 2.1. Korean Peninsula Seismic Time Series

**Figure 1:**
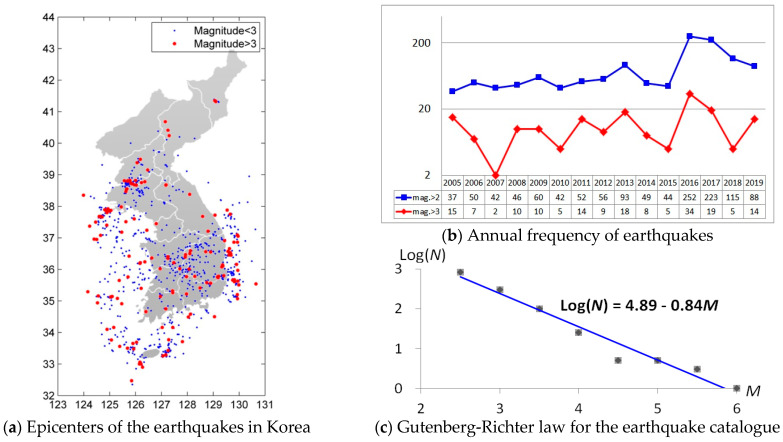
(**a**) Earthquake epicenters around the Korean peninsula, (**b**) annual frequencies of earthquakes for marginal magnitudes 2 and 3 in log−scales, and (**c**) Gutenberg−Richter law for earthquake catalogue: Since the earthquakes of magnitude 5.8 and 5.4 occurred in 2016 and 2017, more than 200 aftershock earthquakes were observed. Additionally, we can see the Gutenberg−Richter law, i.e., the log−scaled frequency is linearly regressed by the Richter magnitude of an earthquake, is satisfied for the Korean peninsula with intercept 4.89 and slope −0.84.

The Korea Meteorological Administration (KMA) has been measuring seismic data since 1 August 1978. Earthquakes have been observed more than 2000 times with a magnitude of 2.0 or greater until 31 May 2020. For four decades, measuring technology has been developed from analogue to digital. Thus, we analyzed only the digital measures since 1 January 2005, for improving the completeness of data. We dealt with 1278 observations consisting of 518 sea areas and 760 inland areas. Figure 1a shows epicenters of the earthquake with a magnitude of 2.0 or more around the Korean peninsula. Earthquakes are mainly occurring in the southern and northwestern inland, southeastern coast, and throughout the western sea. The reason for sporadic earthquake observations in North Korea is that the observations of earthquakes are not easy for geopolitical reasons. However, it is noteworthy that the frequency of seismic observations in the North Korean region is high near Hwang-hae province (38°~39° N, 125°~127° E). Figure 1b shows that the number of observations has been steadily increasing on the Korean peninsula from 2005 to 2020. In particular, the number of earthquakes has increased extensively after the earthquake of magnitude 5.8 in 2016 and 5.4 in 2017. Figure 1c plots the log-scaled frequency versus the magnitude of earthquakes to check the normality of the Korean peninsula earthquake events. We can see the Gutenberg–Richter law, i.e., the log-scaled frequency is linearly regressed by the Richter magnitude of an earthquake, is satisfied for the Korean peninsula with intercept 4.89 and slope −0.84.

### 2.2. Earthquake Network of Grids Linked by Successive Events

Statistical analyses of the seismic time series have been conducted in various ways, focusing on the strength of the earthquake, the occurrence period and so on. Additionally, network analyses on natural and social scientific phenomena are conducted in various ways. Abe and Suzuki analyzed spatiotemporal data of the United States and Japan by their algorithm [32]. The earthquake network of the Korean peninsula in this paper was generated by the following procedures for the Abe and Suzuki methodology.

(1) Partition the considered area into square cells. The grids can have two dimensions using only latitude and longitude, or can have three dimensions using latitude, longitude and depth. Since the number of observations is not so high in the Korean peninsula, two-dimensional grids using latitude and longitude are constructed.
(2) If an earthquake occurs in a partitioned cell, the cell becomes a node of the earthquake network. Even if there are multiple observations in the cell, it cannot be more than one node because the cell was created only by latitude and longitude.
(3) If consecutive earthquakes are observed in two nodes, they are became linked. Then all nodes of the earthquake network are connected like a linear chain. The adjacency matrix of the network is binary, i.e., $A_{ij}=1$ if nodes i and j are connected, and $A_{ij}=0$ otherwise.
(4) Generate random networks as random controls.
  - Shuffle the order of observations and generate the adjacency matrix of the random events.
  - Shuffle the adjacency matrix of the original network.
(5) Iterate the procedure (1)–(4) for various cell sizes.

### 2.3. Network Theory

Network theory is one of the suitable methodologies for explaining complex systems, which is the main field of statistical physics. In addition, network theory is highly versatile, it is widely used in social sciences as well as natural sciences. The network is composed of nodes and links, which is the same as the graph in discrete mathematics. Graph theory has already appeared in the 19th century and has become a field of discrete mathematics. However, network theory in the complex system began to receive attention only after the publishing of the random graph in 1959 by Erdös and Rényi [13], and extensive studies were conducted after the finding small-worldness of complex network in 1998 by Watts and Strogatz [14]. There are numerous network properties to analyze systems or phenomena. In this paper, we investigated some of the most commonly used characteristics in network theory. Detailed definitions of properties were included in Appendix A.

## 3. Results

**Figure 2:**
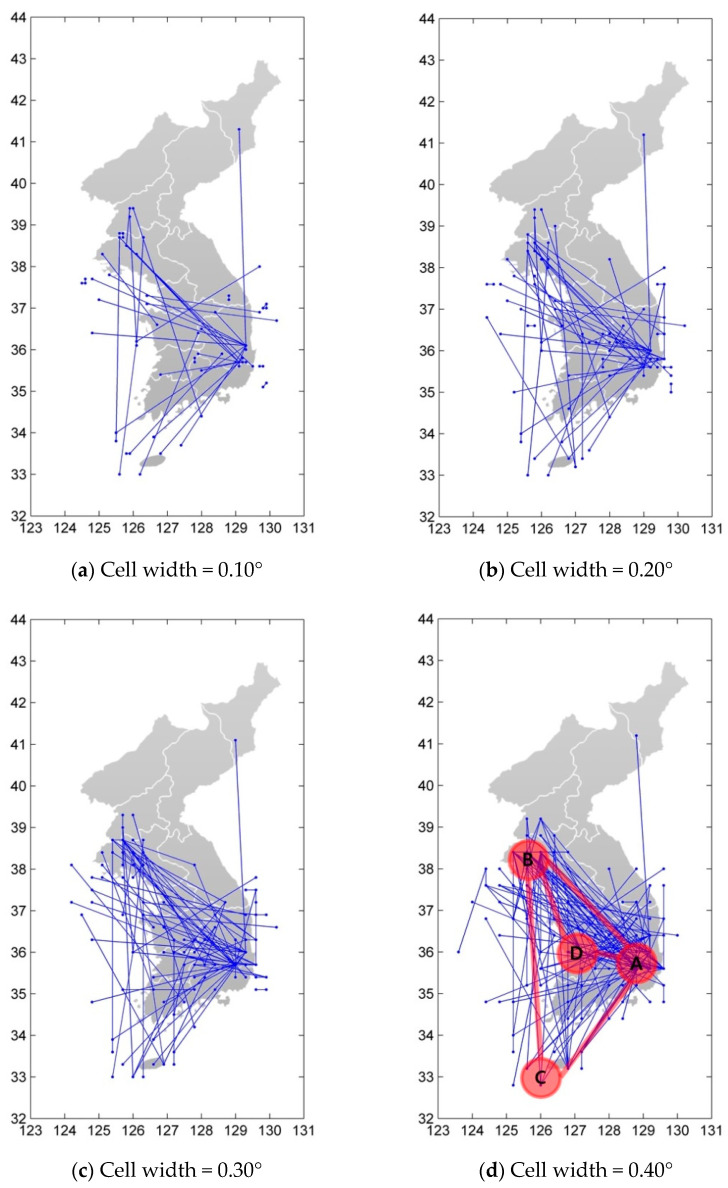
Visualization of the Korean peninsula earthquake network by Abe and Suzuki’s spatiotemporal linkage procedure. Unlike the analyzed network in this research, a link is described only if there are two or more consecutive events between two nodes for visibility. It is described for (**a**) cell width = 0.10°, (**b**) cell width = 0.20°, (**c**) cell width = 0.30°, (**d**) cell width = 0.40°.

In this study, a network was constructed by connecting two nodes if consecutive events were observed between them. Because all grids are connected like a chain in this method, we only showed the nodes and links in Figure 2 when twice or more consecutive events occurred between two grids for visibility. We divide the Korean peninsula into square cells changing their size from 0.01° × 0.01° to 1.00° × 1.00°, and the network was constructed and analyzed.

Earthquakes occurring in a cell are treated as the same node. Therefore, the number of nodes and links decreases as the cell width increases, and the average degree increases. However, the number of nodes does not simply decrease in inverse proportion to the area of the cell. We can easily confirm that hub nodes are formed in Gyeong-ju and Pohang Cities (point A in Figure 2d). This is due to the earthquakes of Gyeong-ju (magnitude 5.8 in 2016) and Pohang (magnitude 5.4 in 2017). Additionally, there are hub nodes in Hwang-hae Province in North Korea (point B in Figure 2d). It is impressive that these two regions are strongly connected to each other. This suggests that there is a strong correlation between the two regions, thus an earthquake may forecast an earthquake of the other regions and prevent severe earthquake damages. As the cell size grows, the connections between epicenters are prominent. We can identify more interesting features in Figure 2d. Gyeong-ju/Pohang (A), Hwang-hae (B), and Jeju (C) make a large triangle, while Gyeong-ju/Pohang (A), Hwang-hae (B) and Chung-cheong (D) make a small triangle. We may cautiously speculate by our earthquake network configurations of the Korean peninsula that the line segments connecting A-B, A-C, B-C, A-D, and B-D are parts of the underground faults.

**Figure 3:**
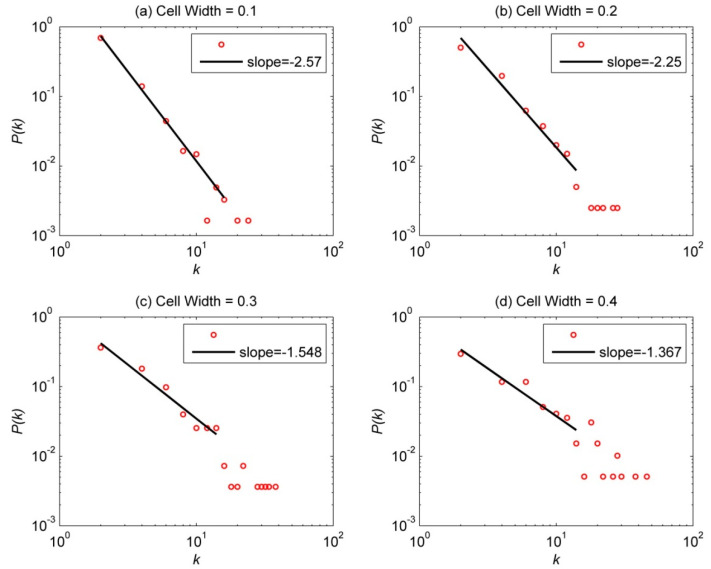
Degree distribution of the earthquake network for 184 months with the magnitude of greater than or equal to 2.0. It is described for (**a**) cell width = 0.10°, (**b**) cell width = 0.20°, (**c**) cell width = 0.30°, (**d**) cell width = 0.40°.

Figure 3 shows the degree distribution for cell widths (a) 0.10° × 0.10° to (d) 0.40° × 0.40°. Here, we only considered the nodes of even degree because of the following reasons. Because the earthquake network has a form like a folded chain in Abe–Suzuki method, the values of the in-degree and out-degree of all nodes are the same in the network except for the start and end points. Hence the most nodes have an even degree. However, if there is a redundancy between two nodes j and k, i.e., the events occur in the order of j, k, and j, the two edges (j, k) and (k, j) created between the two nodes are reduced to one link (j, k). Then the degree of the nodes j and k becomes odd. If there are two redundancies, the degree of the node is even again, and this rule continues. Because the redundancy is rare in general, the degree distribution of nodes including the odd degree shows a zigzag shape. Therefore, we present only even degrees of the distributions and fitted lines to prevent distortions of exponents. In all figures, we can see the degree distributions from power laws. Generally, if the exponent of the degree distribution is less than 3, the variance diverges and the network is called scale-free [33]. That is, $E\left[k^{2}\right]=\int k^{2}P\left(k\right)dk\sim \int k^{2}k^{-\gamma }dk=\int k^{-\left(\gamma -2\right)}dk$ diverges for γ−2<1 by Equation (A1). Therefore, the mean value of the degree cannot be inferred stably because the variance or deviation of the degree diverges infinitely. In almost all papers considering complex networks, the exponent of the degree distribution with power-law distribution is less than 3, so the network with power-law distribution is called a scale-free network. Therefore, it may be judged that the Korean peninsula earthquake network is a scale-free network with an exponent of less than 3. Furthermore, the power exponent is decreasing in the order of 2.570, 2.250, 1.548 and 1.367 as the cell width increases from 0.10° to 0.40°. If the exponent is less than 2, the mean value of a degree, which is the first moment of the degree distribution, is also diverged. Such a network can be called an ultra-scale-free network, and it would be a stronger complex network. Therefore, it is estimated that the earthquake network on the Korean peninsula shows more complex characteristics as the cell width increases.

**Figure 4:**
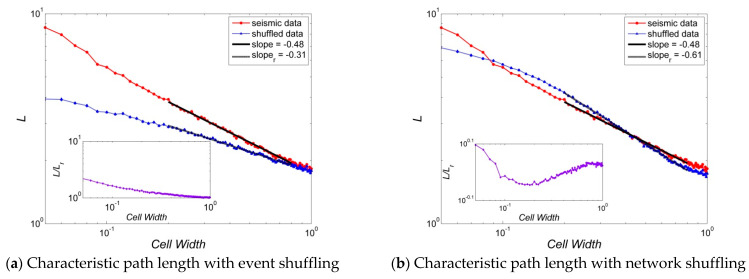
Characteristic path lengths of Korean peninsula earthquake network: Random networks are generated by (**a**) event shuffling, i.e., sequence of order shuffling, and (**b**) network shuffling, i.e., adjacency matrix shuffling. The properties of random data are averages of the shuffled seismic data for 100 times, and all graphs are inserted with an error bar which is 100 times of the standard error, i.e., {r.v.}=m±100∗{s.e.}. Here, the error bars are smaller than the size of the symbols that represent the data points.

**Figure 5:**
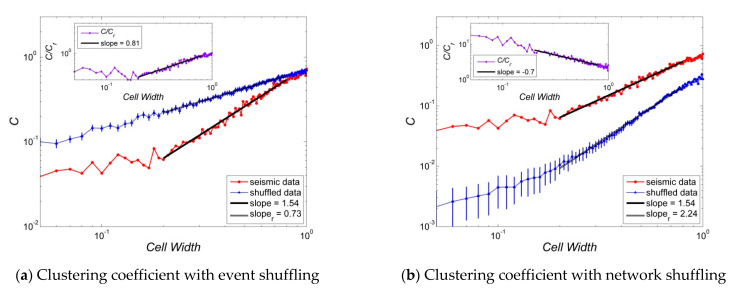
Clustering coefficients of the Korean peninsula earthquake network: Random networks are generated by (**a**) event shuffling, i.e., sequence of order shuffling, and (**b**) network shuffling, i.e., adjacency matrix shuffling. The properties of random data are averages of the shuffled seismic data for 100 times, and all graphs are inserted with an error bar which is 100 times of the standard error, i.e., {r.v.}=m±100∗{s.e.}.

**Figure 6:**
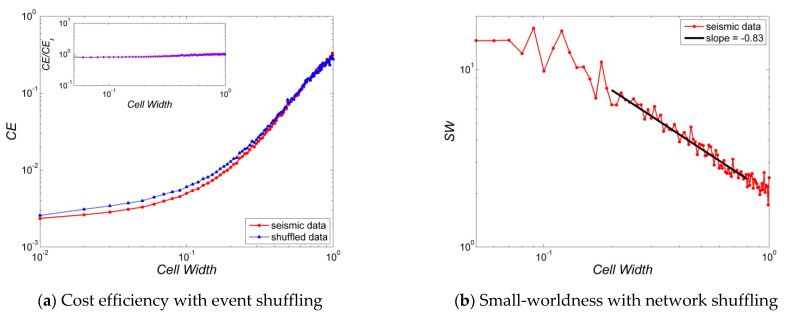
Cost efficiency and Small-worldness of Korean peninsula earthquake network: Random networks are generated by (**a**) event shuffling, i.e., sequence of order shuffling, and (**b**) network shuffling, i.e., adjacency matrix shuffling. The properties of random data are averages of the shuffled seismic data for 100 times, and all graphs are inserted with an error bar which is 100 times of the standard error, i.e., {r.v.}=m±100∗{s.e.}. Here, the error bars are smaller than the size of the symbols that represent the data points.

**Figure 7:**
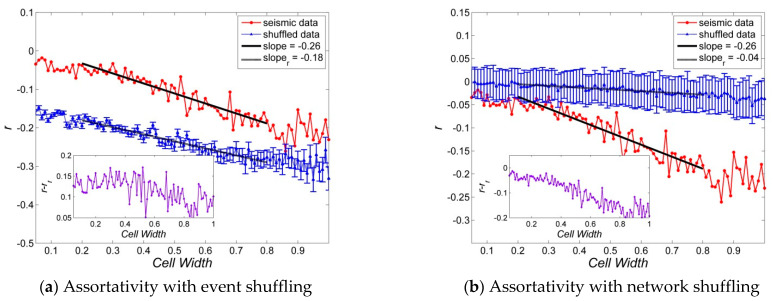
Assortativities of Korean peninsula earthquake network: Random networks are generated by (**a**) event shuffling, i.e., sequence of order shuffling, and (**b**) network shuffling, i.e., adjacency matrix shuffling. The properties of random data are averages of the shuffled seismic data for 100 times, and all graphs are inserted with an error bar which is 100 times of the standard error, i.e., {r.v.}=m±100∗{s.e.}.

**Figure 8:**
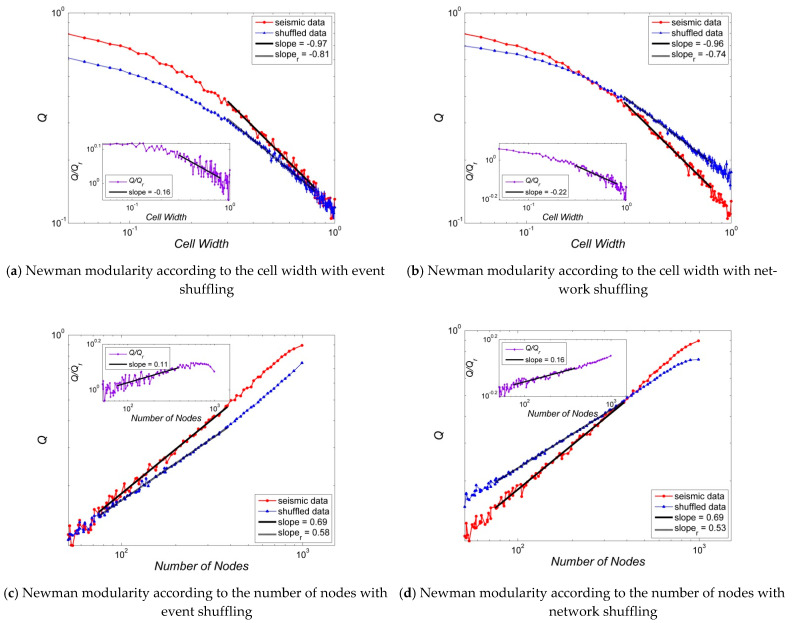
Newman modularities of Korean peninsula earthquake network: Random networks are generated by event shuffling, i.e., sequence of order shuffling ((**a**,**c**)), and network shuffling, i.e., adjacency matrix shuffling ((**b**,**d**)). The properties of random data are averages of the shuffled seismic data for 100 times, and all graphs are inserted with an error bar which is 100 times of the standard error, i.e., {r.v.}=m±100∗{s.e.}. Here, the error bars are smaller than the size of the symbols that represent the data points.

Figure 4, Figure 5, Figure 6, Figure 7 and Figure 8 present several properties of the Korean peninsula earthquake network. The characteristic path length (Figure 4), clustering coefficient (Figure 5), cost efficiency and small-worldness (Figure 6), assortativity (Figure 7), and Newman modularity (Figure 8) are shown. The red solid line presents the values of the actual earthquake network and the blue dashed line presents the values of random networks created by shuffling the order of events and generating the network by Abe and Suzuki’s method (left side of each figure), or only by shuffling the original earthquake network (right side of each figure). In this study, we call EVENT SHUFFLING or SEQUENCE SHUFFLING for the shuffling of the order of event sequence, and NETWORK SHUFFLING or MATRIX SHUFFLING for the shuffling of the adjacency matrix of the earthquake network. We can analyze the properties of Korean peninsula seismic data by comparing them with random events using sequence shuffling. Additionally, it is possible to characterize Korean peninsula seismic data by comparing them with random networks using the matrix shuffling. All axes were presented by log scales except for the assortativity (Figure 7). The number of data points is 95 with their cell widths from 0.05° to 1.00°. All figures show the linearity in the range of the cell width 0.20°~0.80°. That is,

(1) $$r\sim \alpha W\;y\sim W^{\beta }$$

for the cell width W, assortativity r, and other network properties y with a coefficient α and some exponent β.

In the left side of Figure 4, Figure 5, Figure 6, Figure 7 and Figure 8, network properties in linear or log scale show almost identical regularities in both actual and event-shuffled data. Moreover, both properties have the same functional forms with different constant values, i.e., they have identical structures with significantly different parameters. In these graphs, we can compare the effectiveness of actual data with respect to randomly ordered data. On the other hand, on the right side of Figure 4, Figure 5, Figure 6, Figure 7 and Figure 8, the actual and network-shuffled data have similar or heterogeneous functional forms with several cross-over points. We can ordinarily compare the differences between actual networks and random networks using general network theory.

Figure 4 shows the characteristic path length of the earthquake network according to the cell width from 0.05° to 1.00°. In general network analysis, the characteristic path length is expressed as a function of the number of nodes in the network to observe small-worldness. However, the cell width is set as an independent variable in this paper because we analyzed the behavior of the network properties by changing the cell width. The resolution is decreased as the cell width grows. Thus, two points that were previously recognized as different locations are merged as one location, i.e., some nodes are changed to one node as the cell width grows. Therefore, the number of nodes and links, and the average degree in the network decrease as the cell size increases. However, cell width, number of nodes, and average degree are known to have nonlinear relations with each other and do not have a universal property.

The characteristic path length expressed as the log scale shows linearity for the actual data, the event-shuffled data, and the network-shuffled data in the range 0.20°~0.80°. The slopes are −0.48, −0.31, and −0.61, respectively. Thus, as in Equation (1), $L\sim W^{\beta }$ for the characteristic path length L and cell width W with exponents β=−0.48,−0.31, and−0.61.

Figure 4b shows that there are two cross points where the cell widths are 0.09° and 0.40°. The seismic data show that the characteristic path length is shorter than that of the random network for 0.09°~0.40°. This cross-over phenomenon has also been confirmed in previous studies on the United States and Japan [34,35]. However, Japan showed the $L\sim W^{\beta }$ with negative exponents like this study, while the United States showed L~logW.

Figure 5 shows the clustering coefficients referred to both event and network shuffling. Similarly in Figure 4, the clustering coefficient shows linearity in log scaled graph in the range 0.20°~0.80°. Thus, $C\sim W^{\beta }$ for the clustering coefficient C and cell width W with exponents β=1.54, 0.73, and 2.24 for real, event shuffled, and network-shuffled seismic data. Unlike other graphs, cross-over points do not appear in clustering coefficients.

Figure 6 presents the (a) cost efficiency in comparison to event shuffling (sequence shuffling) and (b) small-worldness in comparison to network shuffling (sequence shuffling). As shown in Equation (A5), small-worldness is the ratio of actual network values to random network, so it is only defined for network shuffling. Additionally, as expressed in Equation (A6), cost efficiency is the ratio of the number of links to all possible links. Thus, comparing two networks is not meaningful because the number of links is invariant for network shuffling. In Figure 6a, the cost efficiency (CE) is an S-shaped curve in log scale, but has almost linearity in the range of 0.20°~0.80°. Thus, $CE\sim W^{\beta }$ for the cost efficiency CE and cell width W with the exponents β=2.22 and 2.08 for actual and event-shuffled seismic data. Interestingly, CE of actual and event-shuffled data have almost the same functional forms with a nearly constant ratio of 0.81~1.06. On the other hand, the magnitude of CE has a range of 0.01~0.2 for the range 0.20°~0.80° of cell width. This is similar to United States (0.03~0.3), but is far from Japan (0.002~0.03) [34,35]. The reason for this phenomenon is presumed to be that the number of repeated links is reduced to one in the Japanese earthquake network with numerous earthquakes. Since our network generates a node only if an event occurs, the low CE value can be interpreted as the high repeatability of links, not simply the low number of links. In general, there is a close relationship between CE (cost efficiency), L (characteristic path length), and C (clustering coefficient). If a network has a high-cost efficiency, i.e., if the number of links is high, the compactness of the network increases and the capability of generating triangles increases, so the clustering coefficient increases. Additionally, if there are many triangles, the capability of a direct path from one node to the other without passing through another node increases. For instance, consider a triplet (i, j, k) in a network, then the path length from i to k is 2. However, it is reduced to 1 if the triplet is in fact a triangle so that a direct path from i to k exists. Therefore, efficiency leads to high clustering coefficient and low characteristic path length in general. However, the Korean peninsula earthquake network has a large CE (84~103 times) despite having higher L and lower C than the event-shuffled network. This is presumed to be a crucial property of a chain-like connected earthquake network.

In Figure 6b, small-worldness (SW) in log scale has linearity in the cell size of 0.20°~0.80° and the slope is −0.67. Thus, $SW\sim W^{\beta }$ for the small-worldness SW and cell width W with the exponent β=−0.83. Now for the value, SW has a range of 2.19~6.33 with the domain of cell size 0.20°~0.80°. Generally, a network is called to be a small-world network if $C\gg C_{\mathrm{rand}}$ for $L≅L_{\mathrm{rand}}$ so $SW\equiv \frac{C/C_{\mathrm{rand}}}{L/L_{\mathrm{rand}}}\gg 1$. According to this criterion, the Korean earthquake network has a weak small-worldness. In previous studies, small-worldness in the United States (about 3~20) was similar to this study and that of Japan (about 12~105) was larger than this study [34,35]. In fact, the small-worldness is mainly dependent on the number of nodes.

Figure 7 shows the assortativities compared to sequence and network shuffling on a linear scale. The assortative coefficients (r) of actual and event-shuffled seismic data have linearity in the cell size of 0.20°~0.80° and its indices are −0.26 and −0.18. Thus, as shown in Equation (1), r~αW for the assortative coefficient r and cell width W with the coefficients α=−0.26 and−0.18 for actual and event-shuffled seismic data. Interestingly, the difference between r of real and event-shuffled data remains nearly constant in the range of 0.05~0.17 except for variations in fluctuation levels. General networks have weak negative correlations, and the Korean earthquake network also has a weak negative correlation of −0.23~−0.05. However, in Figure 7a, event-shuffled data has a −0.29~−0.17 range, and has a significantly strong negative correlation coefficient compared to actual seismic data. In Figure 7b, The assortative coefficient of the actual network is significantly larger than the shuffled network data for the range of 0.40°~1.00°. Absolute values of the assortative coefficients are sorted as event-shuffled network, actual network, and matrix shuffled network in order. Therefore, we can infer that the Korean peninsula earthquake network has a relatively positive correlation compared to the event-shuffled network. However, the assortativity of the actual network is also significantly low compared to the matrix shuffled network.

Finally, Figure 8 shows the modularity that presents the community structure of the Korean peninsula earthquake network on the log scale. Modularity is expressed not only versus the cell width but also versus the number of nodes because regularities are relatively clearer in both independent variables than the other properties in this paper. The modularity of the Korean peninsula earthquake network shows a decreasing manner for cell size, and of course, the increasing pattern for the number of nodes. The shape is similar to general earthquake networks. However, the modularity is not large enough, so it is difficult to judge whether the network is modular or not. As expressed in other figures, the linearity of the modularity is also prominent in the domain of 0.20°~0.80°. Thus, $Q\sim W^{\beta }$ for the Newman modularity Q and cell width W with exponents β=−0.97,−0.81, and−0.74 for actual, event shuffled, and network-shuffled seismic data. Additionally, $Q\sim {\left[N\left(W\right)\right]}^{\beta }$ for the Newman modularity Q and the number of nodes N(W) according to the cell width W with exponents β=0.69, 0.58, and 0.53 for actual, event shuffled, and network-shuffled seismic data. The linearity is clearer when using the number of nodes instead of the cell width as independent variables. The sub-graphs that express the ratio of actual data to shuffled data in a small inset show the global linearity more clearly ((c) and (d)). Similar to other network properties, there are significant differences between real and event-shuffled data ((a) and (c)), while cross points exist between real and matrix-shuffled data ((b) and (d)). The cross-over points occur where the cell width is 0.20°, and the number of nodes N(W) is 401.

Table 1 is a summary of the network properties described in Figure 4, Figure 5, Figure 6, Figure 7 and Figure 8. That is, the total number of nodes, average degree, characteristic path lengths, clustering coefficient, cost efficiency, small-worldness, assortativity, and modularity of actual data, event (sequence) shuffled data, and network (matrix) shuffled data.

## 4. Discussion

The Korea Meteorological ministration (KMA) has been measuring seismic data since 1 August 1978, and has made observations more than 2000 times with a magnitude of 2.0 or more up until 31 May 2020. Among them, we dealt with the 1278 observations since 1 January 2005. The number of earthquakes has slightly increased in the Korean peninsula for the past 15 years (Figure 1).

We partitioned the considered area into square grids and counted it as a node if an earthquake occurred in a grid. Then, we generated a network using the Abe and Suzuki method, i.e., a link of the network is made if successive events occurred between two nodes. We analyzed the properties of the Korean peninsula earthquake network and performed a significance test by comparing the network properties computed from two random control methods: (a) event (sequence) shuffled data as randomizing the order of events, and (b) network (matrix) shuffled data as randomizing the adjacency matrix of original earthquake network. Sequence-shuffled networks showed a clear difference with the Abe–Suzuki earthquake networks although both of them have the same functional form, indicating the statistical significance of network properties of the Korean earthquake network (left side of Figure 4, Figure 5, Figure 6, Figure 7 and Figure 8). This result supports the spatiotemporal causality in Abe–Suzuki-based earthquake networks. As for the matrix-shuffled random networks, they have a somewhat different functional form with the Abe–Suzuki earthquake network, showing several cross-over points (right side of Figure 4, Figure 5, Figure 6, Figure 7 and Figure 8). The two shuffled networks exhibited completely different functions, and one is even significantly higher, but the other is lower compared to the real Korean Abe–Suzuki network. For most cases, the event-shuffled network showed a functional similarity to the real network. This different behavior requires a criterion to be established when generating random networks associated with a target network for a nonparametric significance test. Furthermore, the Korean earthquake network shows strong linearity in the cell width range of 0.20°~0.80°, which can be used as a reference for studies to find inherent regularities and forecast an earthquake.

As for network properties of the Korean earthquake network, the degree distribution seems to show a power-law behavior with varying exponents depending on cell sizes; increasing cell sizes leads to smaller power exponent and stronger scale-free property (Figure 3). When compared to random networks, clustering coefficients have a larger value although with a similar average path length (Figure 4 and Figure 5). In addition, the Korean earthquake network has a weak small-worldness (Figure 6) and a strong scale-free network. As for the network assortativity, the Korean earthquake network has a negative coefficient when the cell width exceeds 0.20° (Figure 7). Lastly, the network modularity shows a decreasing and increasing behavior with the cell width and the number of nodes, respectively (Figure 8).

## 5. Conclusions

In this work, we presented the significance of network characteristics of the Korean peninsula earthquake network by comparing it with randomly generated networks. Examining earthquake network properties generated by Abe–Suzuki method over varying cell sizes from 0.01° × 0.01° to 1.00° × 1.00°, a strong dependency between them implies the presence of the most optimized cell structure. We confirmed that regularity and linearity are well pronounced in a specific range of cell widths. This finding supports the presence of the most predictable grid structure which makes the analysis of earthquakes easier. In the Korean peninsula case, the network properties show strong linearity when the cell size is within the regime of 0.20°~0.80°. Additionally, it is worth noting that connectivity in earthquake networks can be strongly related to geological fault structure.

We introduced the *EVENT* (*SEQUENCE*) *SHUFFLING* and compared it with the most used *NETWORK* (*MATRIX*) *SHUFFLING*, and then we found that some network properties are significantly higher than a shuffled network, but lower than the other shuffled network. This suggests that the carefulness of network interpretation is necessary. Since the network shuffling makes the number of nodes and links unvaried with respect to the actual network, it may not be interpreted as the pure shuffling of the original event data. On the other hand, the event-shuffled network may not be a pure random network, i.e., it may show some regular network properties.

Therefore, we propose the following criterion:

(1) To see the characteristics of the earthquake network, it is better to compare it with *NETWORK (MATRIX) SHUFFLING*.
(2) To see the characteristics of the earthquake event via network analysis, it is more convenient to compare it with *EVENT (SEQUENCE) SHUFFLING*.

In the future, we will consider the statistical meaning of earthquake networks by constructing and analyzing more diverse types of networks (e.g., visibility network). We expect that the statistical inference will help to improve the forecasting ability of the earthquakes.

## Figures and Tables

**Table 1:** Network properties of earthquake network of the Korean peninsula with respect to event (sequence) shuffled data and network (matrix) shuffled data.

Cell Width	0.10°	0.20°	0.30°	0.40°	0.50°	0.60°	0.70°	0.80°	0.90°	1.00°
N	610	401	276	197	143	111	90	71	62	52
〈k〉	3.04	4.33	6.12	7.97	10.13	12.11	13.44	15.1	15.23	14.27
L	5.56	3.91	3.18	2.75	2.43	2.24	2.11	1.94	1.91	1.83
$L_{\mathrm{seq}}$	3.4	2.89	2.58	2.4	2.2	2.09	2	1.89	1.85	1.79
$L_{\mathrm{mat}}$	5.74	4.22	3.3	2.76	2.39	2.13	1.96	1.81	1.77	1.73
C	0.04	0.06	0.11	0.16	0.27	0.32	0.43	0.5	0.59	0.73
$C_{\mathrm{seq}}$	0.14	0.23	0.31	0.38	0.44	0.49	0.52	0.6	0.66	0.7
$C_{\mathrm{mat}}$	0.004	0.011	0.022	0.041	0.071	0.11	0.15	0.22	0.25	0.28
CE	0.005	0.011	0.022	0.041	0.071	0.11	0.15	0.22	0.25	0.28
$CE_{\mathrm{seq}}$	0.006	0.013	0.025	0.044	0.075	0.11	0.15	0.21	0.25	0.28
SW	9.81	6.33	5.32	3.92	3.76	2.78	2.64	2.19	2.18	2.46
r	−0.03	−0.04	−0.03	−0.07	−0.1	−0.12	−0.15	−0.18	−0.15	−0.23
$r_{\mathrm{seq}}$	−0.17	−0.2	−0.2	−0.23	−0.25	−0.26	−0.25	−0.28	−0.28	−0.33
$r_{\mathrm{mat}}$	−0.004	−0.004	−0.011	−0.009	−0.014	−0.02	−0.029	−0.028	−0.036	−0.037
Q	0.68	0.5	0.37	0.28	0.25	0.19	0.16	0.13	0.13	0.13
$Q_{\mathrm{seq}}$	0.52	0.39	0.31	0.25	0.21	0.18	0.17	0.14	0.12	0.12
$Q_{\mathrm{mat}}$	0.62	0.49	0.39	0.33	0.28	0.24	0.22	0.19	0.18	0.18

N: total number of nodes 〈k〉: average degree (average number of links); L: characteristic path length; C: clustering coefficient; CE: cost efficiency; SW: small-worldness; r: assortativity; Q: modularity. ${\left\{*\right\}}_{\mathrm{seq}}$: property of event (sequence) shuffled data. ${\left\{*\right\}}_{\mathrm{mat}}$: property of network (matrix) shuffled data.

## Data Availability

Not applicable.

## Appendix A

### Appendix A.1. Degree Distribution

Degree distribution is one of the most accurate measurements of the scale-free property of a network. The number of links connected to each node in the network is called degree, and the distribution of these values is called degree distribution. Over the past few decades, studies on networks have been radically conducted. Many networks are complex and most of these complex networks have scale-free properties. In other words, the distributions of each degree k of the most complex networks show power-law with exponents γ less than or equal to 3.

(A1) $$P\left(k\right)\sim k^{-\gamma }$$

If the exponent of the degree distribution is less than 3, the second moment diverges. Thus the network is called a scale-free network.

### Appendix A.2. Characteristic Path Length

Characteristic path length is the average value of distances between any two nodes in the network. If we define $L_{ij}$ as the length of the shortest path, i.e., distance between node i and j, the characteristic path length is defined as follows.

(A2) $$L=\frac{2}{N\left(N-1\right)}\overset{N}{\underset{i=1}{\sum }}\overset{i-1}{\underset{j=1}{\sum }}L_{ij}$$

In the above equation, $L_{ij}$ is summed except for infinity. It is because $L_{ij}$ becomes infinity and L also becomes infinity if we sum $L_{ij}$ of two disconnected nodes, so the characteristic path length becomes meaningless. If L is proportional to or less than logN, the network is called to be a small-world network.

### Appendix A.3. Clustering Coefficient

Let $k_{i}$ be the degree of node i and $\Delta_{i}$ be the number of triangles having node i as a vertex. Any two selections among $k_{i}$ links form a triplet, say triplet of j−i−k. If the other two nodes j and k that make up the triplet are connected, the triplet will create a triangle. The probability of creating a triangle with respect to any selection of triplets around a node i is defined as follows.

(A3) $$C_{i}=\frac{\mathrm{number}\;\mathrm{of}\;\mathrm{triangles}}{\mathrm{all}\;\mathrm{possible}\;\mathrm{number}\;\mathrm{of}\;\mathrm{triplets}}=\frac{\Delta_{i}}{k_{i}\left(k_{i}-1\right)/2}$$

for the neighborhood of node i.

The average value of these $C_{i}$ is called the clustering coefficient.

(A4) $$C=\overset{N}{\underset{i=1}{\sum }}C_{i}$$

the clustering coefficient is also used to obtain insight of the small-world property or the aggregation of links [27].

### Appendix A.4. Small-Worldness

From the relation between the characteristic path length L and the clustering coefficient C mentioned above, we can confirm whether the network satisfies the small-world property. Let L and C be the characteristic path length and clustering coefficient of a given network, and $L_{\mathrm{rand}}$ and $C_{\mathrm{rand}}$ be the properties of a random network. Then the small-world property is calculated as follows.

(A5) $$SW=\frac{C/C_{\mathrm{rand}}}{L/L_{\mathrm{rand}}}$$

generally, a network is called to have the small-world property if $C\gg C_{\mathrm{rand}}$ for $L≅L_{\mathrm{rand}}$ so $SW=\frac{C/C_{\mathrm{rand}}}{L/L_{\mathrm{rand}}}\gg 1$.

### Appendix A.5. Cost Efficiency

The cost of logistics or transferring information between any two nodes depends on how many different links exist between the two nodes. Hence, cost efficiency is defined as the ratio of the number of links in the actual network to those of the complete fully connected network. Let n and l be the number of nodes and links in a network. Then, the cost efficiency is defined as follows.

(A6) $$CE=\frac{\mathrm{number}\;\mathrm{of}\;\mathrm{links}}{\mathrm{all}\;\mathrm{possible}\;\mathrm{number}\;\mathrm{of}\;\mathrm{links}}=\frac{l}{n\left(n-1\right)/2}$$

### Appendix A.6. Assortativity

Assortative network tends to connect rich nodes (many links) to rich nodes, or poor nodes (a few links) to poor nodes. Thus the assortativity is derived by the following [36].

(A7) $$r=\frac{1}{\sigma_{q}^{2}}\underset{j,\;k}{\sum }jk\left(e_{jk}-q_{j}q_{k}\right)$$

where $q_{j}\equiv \left(j+1\right)p_{j+1}/\overset{N}{\underset{i=1}{\sum }}ip_{i}$ is the probability that a remaining degree is j. Here, $p_{i}$ is the probability of the degree distribution such that the number of links is i for a node. Additionally, $e_{jk}$ presents the joint probability for remaining degrees of j and k. Meanwhile, $\sigma_{q}^{2}$ is the product of the standard deviations for remaining degrees of j and k. Since the two distributions are identical and independent, it can be expressed as the variance of the degree j. As a result, assortativity has a value between −1 and 1 because it is the correlation coefficient of the remaining degree distribution. If a network is assortative, the assortativity exhibits a positive number.

In the co-authors’ networks of mathematical or scientific journals, film actors, and executive networks of companies, the assortativity has a positive value of 0.1 to 0.3. The world-wide web, protein–protein interaction, neural network, and food chain are known to have negative values of −0.1 to −0.3. On the other hand, the amino acids interaction network in a protein has a positive value of 0.1 to 0.5 despite that the proteins interaction network has a negative value of −0.2. In other words, the assortativities exhibit differences depending on the resolution even though they are calculated on the same [19].

### Appendix A.7. Modularity

Modularity is one of the measurements to identify the community structure of a network. If a network has N nodes, the modularity is defined as follows [37,38].

(A8) $$Q=\frac{1}{2m}\overset{N}{\underset{i,\;j=1}{\sum }}\left(A_{ij}-\frac{k_{i}k_{j}}{2m}\right)\delta \left(c_{i},\;c_{j}\right)$$

Here, $k_{i}$ and $k_{j}$ are degrees for nodes i and j, m is the total number of links, and $\delta \left(c_{i},\;c_{j}\right)$ is the Kronecker delta function with a value of 0 or 1. $\delta \left(c_{i},\;c_{j}\right)=1$ if nodes i and j are contained to the same community, and $\delta \left(c_{i},\;c_{j}\right)=0$ otherwise. The community or clustering structure of a network is decided by Newman algorithm. $A_{ij}$ is the actual number of connections with the value of 0 or 1, and $k_{i}k_{j}/2m$ is the probability of connections between nodes i and j. Then, the range of the modularity for unweighted and undirected networks lies in the range [−1/2, 1]. In general, it is judged that the separation between the groups is good if the modularity is 0.3 or more.
